# The Effect of Inferior Oblique Muscle Z-Myotomy in Patients with Primary Inferior Oblique Overaction

**DOI:** 10.4274/tjo.galenos.2019.87947

**Published:** 2020-04-29

**Authors:** Hasan Kızıltoprak, Hakan Halit Yaşar, Kemal Tekin

**Affiliations:** 1 Bingöl Women’s Health and Children’s Hospital, Department of Ophthalmology, Bingöl, Turkey; 2 Ulucanlar Eye Training and Research Hospital, Clinic of Ophthalmology, Ankara, Turkey; 3 Erciş State Hospital, Clinic of Ophthalmology, Van, Turkey

**Keywords:** Inferior oblique muscle overaction, surgical results, Z-myotomy

## Abstract

**Objectives:**

To investigate the surgical results of the inferior oblique muscle Z-myotomy in patients with inferior oblique muscle overaction (IOOA).

**Materials and Methods:**

The medical records of patients who had undergone inferior oblique muscle Z-myotomy for primary IOOA in a single center between 2017 and 2018 were retrospectively analyzed. All patients had mild IOOA (+1 and between +1 and +2). Preoperative and postoperative IOOA degrees and ocular motility examinations were evaluated. Inferior oblique muscle Z-myotomy is performed at 6 mm along the physiological muscle line after identifying the lower oblique muscle through an inferotemporal fornix incision.

**Results:**

Forty-seven eyes of 44 patients were included in the study. The patients were divided into those with +1 IOOA (n=37, 78.7%) and those with +1-2 IOOA (n=10, 21.3%). The mean age of the +1 group was 14.18±11.8 years and the mean age of the +1-2 group was 13.40±7.45 years. The mean follow-up time was 10.56±8.7 (6-17) months. Bilateral Z-myotomy was performed in 3 (6.8%) and unilateral in 41 (93.2%) of the patients. IOOA correction was observed in 43 (91.4%) of the 47 eyes after Z-myotomy, while 4 (8.6%) eyes still had preoperative levels of IOOA. There was no statistically significant difference in surgical success rate between the groups (p=0.849). When preoperative and postoperative IOOA values were compared, there was a statistically significant decrease in IOOA values in the postoperative period (p=0.001). No intraoperative or postoperative complications were observed.

**Conclusion:**

Inferior oblique Z-myotomy is a simple, fast, sutureless surgical procedure in which the original muscle insertion is preserved. Z-myotomy of the inferior oblique muscle can be used as a successful attenuation method in patients with minimal IOOA.

## Introduction

Inferior oblique muscle overaction (IOOA) is a common eye movement disorder characterized by excessive elevation of the adducted eye.^1^ IOOA is etiologically and clinically evaluated under two headings, primary and secondary.^2^ Primary IOOA is a condition of unknown etiology and is not associated with paralysis of any muscle. Secondary IOOA occurs as a result of paralysis of the superior oblique or contralateral superior rectus muscle of the same eye.^3^

In IOOA, the diplopia that occurs due to hyperdeviation and the unaesthetic upward deviation of the eye on adduction make the patient uncomfortable and are an indication for surgery.^3,4^ Regardless of etiological differences, surgical treatment of both primary and secondary IOOA is based on the principle of reducing muscle function. Depending on the surgeon’s experience and preference, the procedures most commonly used in the operation are inferior oblique muscle recession, anteriorization, tenotomy, and myectomy.^5,6,7,8^ In patients with low-grade (between +1 and +1-2) IOOA, one alternative is Z-myotomy, an extraocular muscle weakening procedure performed by making two incisions in the longitudinally opposing margins of an extraocular muscle.^9^ The few published articles on the results of Z-myotomy have reported positive outcomes.^9,10^

The objective of this study was to evaluate the effectiveness of Z-myotomy surgery performed at our clinic to correct isolated primary IOOA.

## Materials and Methods

The study protocol was approved by the Ankara Numune Training and Research Hospital Ethics Committee and the study was carried out in accordance with the Declaration of Helsinki. The medical records of patients who underwent Z-myotomy for primary IOOA at Ulucanlar Ophthalmology Training and Research Hospital between January 2017 and June 2018 were evaluated retrospectively. As per routine protocol, the possible risks of surgery were explained to patients and informed consent forms were obtained preoperatively.

**Figure 1:**
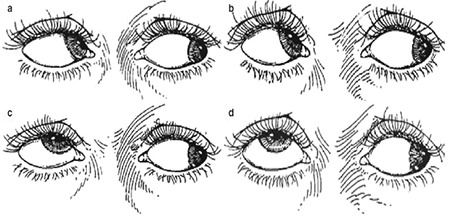
Assessment of inferior oblique overaction severity. When the fixated eye is in abduction, 5° of excessive elevation of the eye in adduction was evaluated as +1 (a), 10° as +2 (b), 15° as +3 (c), and 20° as +4 (d)

**Figure 2:**
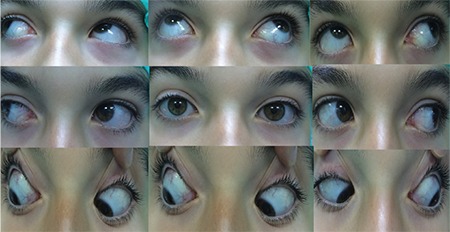
Preoperative appearance of a patient with bilateral IOOA and V pattern

After history was taken, all patients underwent ophthalmologic examination including detailed strabismus examination. All patients were evaluated pre- and postoperatively for presence of A-V pattern and horizontal and vertical deviations with and without glasses. As described previously by Del Monte and Parks^11^, IOOA was graded as +1, +2, +3, and +4 for angles of excess elevation of the adducted eye of 5°, 10°, 15°, and 20°, respectively, when the fixating eye was at 30 degrees of abduction and 20 degrees of elevation. This classification is shown in Figure 1.^12^ Surgical indication was determined for patients with minimal IOOA but significant V pattern (>15 PD), bilateral cases with asymmetric deviation of one eye between +1 and +1-2, and patients who requested IOOA below +2 to be treated during horizontal surgery. Z-myotomy surgery was planned for patients who had IOOA meeting these criteria (between +1 and +1-2). None of the patients included in the study had history of any previous eye surgery. Figure 2 shows the preoperative appearance of a patient with bilateral IOOA and V pattern.

**Figure 3:**
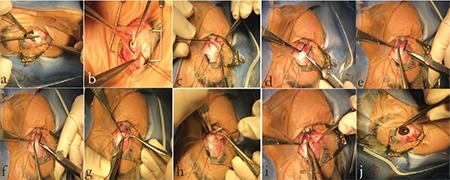
In Z-myotomy, the inferior oblique muscle is accessed via inferotemporal fornix incision, isolated with a hook, and stretched between two hooks (a, b, c). Two clamps are placed at opposite ends of the muscle to cover approximately 75% of the muscle belly and isolate the middle 50% of the muscle belly between the two clamps (d). The first clamp is placed 7-8 mm from the muscle insertion and the second clamp 10 mm from the first clamp (d). Using Wescott scissors, the muscle is cut from the margin to the middle of its horizontal section at two points 4-6 mm apart in the direction of blood flow from the clamps (closer to the insertion) to optimize hemostasis (e-i). The cut muscle margins are cauterized using lowtemperature heat cautery. The conjunctiva is closed with individual 8.0 sutures (j)

In the Z-myotomy technique, the inferior oblique muscle was accessed via inferotemporal fornix incision, isolated with a hook, and stretched between two hooks (Figures 3a-c). Two clamps were placed at opposite ends of the muscle so as to cover approximately 75% of the muscle belly. Thus, the middle 50% of the muscle belly was isolated between the two clamps (Figure 3d). The first clamp was placed 7-8 mm from the muscle insertion and the second clamp 10 mm from the first clamp (Figure 3d). Using Wescott scissors, the muscle was cut from the margin to the middle of its horizontal section at two points 4-6 mm apart in the direction of blood flow from the clamps (closer to the insertion) in order to optimize hemostasis (Figure 3e-i). The cut edges of the muscle were then cauterized using low-temperature heat cautery. The conjunctiva was closed with individual 8.0 sutures (Figure 3j). An epinephrine and lidocaine mixture was administered by sub-Tenon’s injection, and topical antibiotic and anti-inflammatory agents were instilled. All operations were performed by a single surgeon (H.H.Y.).

Patients were followed-up at 1 day, 1 week, 1 month, and 6 months after surgery, and every 6 months thereafter. Criteria for success were complete elimination of IOOA and resolution of the hyperdeviation upon inferior oblique movement. Persistence of hyperdeviation in primary gaze and IOOA on adduction was considered failure.

### Statistical Analysis

In statistical analysis, arithmetic mean ± standard deviation values were calculated for each variable. Surgical success was compared between the groups (+1/+1-2) using chi-square test. A p value less than 0.05 was considered statistically significant.

## Results

Forty-seven eyes of 44 patients were evaluated in the study. Of the patients included in the study, 26 (59%) were male and 18 (41%) were female. The patients were divided into two groups, the +1 group and the +1-2 group. IOOA grade was +1 in 37 patients (78.7%) and between +1 and +2 in 10 patients (21.3%). Mean age was 14.18±11.8 (4-55) years in the +1 group and 13.40±7.45 (5-33) years in the +1-2 group. The mean follow-up time of the patients was 10.56±8.7 (6-17) months. Three patients (6.8%) underwent bilateral Z-myotomy and 41 (93.2%) underwent unilateral Z-myotomy.

**Figure 4:**
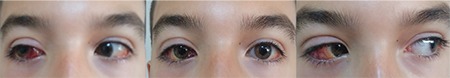
correction is observed in postoperative examination of the patient shown in Figure 2 IOOA: Inferior oblique muscle overaction

IOOA improved after surgery in 43 (91.4%) of the 47 eyes that underwent Z-myotomy, while IOOA remained at the preoperative level in 4 (8.6%) eyes. Of the 4 patients with failed surgery, 3 were in the +1 group and 1 was in the +1-2 group. Postoperative success rate did not differ significantly between the groups (p=0.849). Mean preoperative and postoperative IOOA grades were +1.15±0.13 and 0.12±0.20, respectively (p=0.001). No intra- or postoperative complications were observed. Figure 4 shows postoperative correction of IOOA in the patient whose preoperative image is shown in Figure 2.

## Discussion

Treating small-angle but symptomatic hypertropias is challenging. Because surgical methods performed in these patients (inferior oblique muscle recession, anteriorization, tenotomy, and myectomy) can result in excessive or insufficient correction, satisfactory outcomes may not always be achieved. There have been few publications to date on the effect of Z-myotomy of the inferior oblique muscle performed for mildly symptomatic IOOA. The results of our study demonstrated that Z-myotomy performed as surgical treatment for mild IOOA is quite successful.

The main goal of procedures used in the treatment of IOOA, such as recession, anteriorization, tenotomy, myectomy, and muscle extirpation-denervation, is to weaken the inferior oblique muscle.^13,14^ Superiority studies comparing these methods have shown that all are similarly effective and no method is significantly superior to the others.^15^

After tenotomy or myectomy, the inferior oblique muscle may exhibit random adhesion to the sclera in the retroequatorial region, which can cause excessive or insufficient correction.^8,16,17^ In revision operations performed to treat this excessive or insufficient correction, the inferior oblique muscle cannot be isolated again as a whole single muscle due to the fibers adhering at different places to the sclera, and a substantial proportion of revision operations fail for this reason.^8,13,17^ Based on our observations, excessive or insufficient correction is relatively less frequent with recession and anteriorization because the inferior oblique muscle is sutured adjacent to the insertion of the inferior rectus muscle. Furthermore, the fact that the muscle is whole and in a known position facilitates reisolation of the muscle and recorrection in patients undergoing revision due to excessive in insufficient correction. Therefore, recession or anteriorization are preferred over tenotomy or myectomy.^13,18^ However, in revision operations performed after these methods, it is observed that the inferior oblique muscle is surrounded by a denser fibrotic tissue compared to previously recessed horizontal muscles.^17^ This may cause difficulty separating the muscle from the sclera in some cases. Because these methods can also result in excessive or insufficient correction and due to the aforementioned difficulty in revision operations, the Z-myotomy method (in which the inferior oblique muscle is not detached from its insertion) may be an alternative to tenotomy and myectomy for mild cases of IOOA.

The practice of performing Z-myotomy on the inferior oblique muscle was introduced in 1973 by De Decker and Kueper^19^ with a technique similar to that used today.Mellott et al.^20^ later performed this procedure on 10 patients with mild IOOA and achieved successful outcomes.Similar results were also reported in subsequent studies.

Inferior oblique Z-myotomy has become particularly popular in the treatment of mild IOOA in recent years. This technique has many advantages over other methods.^9,10^ Inferior oblique Z-myotomy is a short surgical procedure that is relatively easy to perform. As it does not require any suturing to the sclera, the risk of scleral perforation or vortex vein damage is low. In addition, the original insertion of the muscle is not altered in this surgery.^9,10,21,22^ It was recently emphasized that inferior oblique Z-myotomy is a technically easy, complication-free, and effective surgical procedure for patients with primary and secondary IOOA.^9,10^ We achieved a 91.4% success rate in patients with mild but symptomatic primary IOOA who underwent Z-myotomy and were followed in our center. In 8.6% of our patients, the same degree of IOOA persisted after surgery. Excessive correction or other complications did not occur in any of our patients. No recurrence was observed during the follow-up period of approximately 6 months. These results demonstrate the success of this procedure when used to treat small-angle deviations.

Z-myotomy or marginal myotomy can also be performed on the horizontal rectus muscles. While this method is occasionally preferred for small-angle horizontal deviations as well, its main indication is for achieving extra attenuation in maximally recessed horizontal rectus muscles. The muscle is weakened by reducing the number of contractile elements in the muscles without changing their arc of contact with the globe. Other indications for the practice of this method on the rectus muscles include eyes with an extremely thin sclera, implants, explants, and cerclage bands placed after retinal detachment surgery.^23,24^

It has been reported that inferior oblique Z-myotomy can also be safely used in the treatment of patients with accompanying horizontal deviations.^9,15^ In such cases, it was stated that Z-myotomy can be performed to correct small-angle IOOA accompanying horizontal deviations and that there is no need to change the planned horizontal deviation surgery.^9,25^ Because our study was conducted on patients with isolated IOOA, the effect of Z-myotomy on horizontal deviation could not be evaluated.

Moreover, in asymmetric bilateral cases, successful outcomes were also reported with inferior oblique Z-myotomy performed on the side of lower severity.^9,13^ In our series, all 3 patients with bilateral IOOA showed asymmetry. We performed bilateral Z-myotomy for this reason and observed successful correction of the IOOA on both sides. Therefore, we can conclude that bilateral inferior oblique Z-myotomy is also successful in mild cases with different degrees of IOOA.

The main limitations of this study are the retrospective design and the fact that we did not make a comparison with other surgical procedures. In addition, our study group was small and for this reason we cannot generalize the results. However, inclusion of only patients with isolated IOOA enabled a better assessment of the outcomes of this procedure and is the strength of our study.

## Coclusion

In conclusion, inferior oblique Z-myotomy is an easy-to-perform, complication-free, successful surgical technique for the treatment of patients with low-grade (between +1 and +1-2) IOOA. For small-angle but symptomatic cases, this method may be preferable to recession and myectomy, which can lead to excessive correction. The effect of changing the distance of incisions made in the inferior oblique muscle, the distance between incision and insertion, or the depth of the incision in lower or higher degree deviations during this surgical procedure may inspire future studies.
